# Pharmacometabolomics of trabectedin in metastatic soft tissue sarcoma patients

**DOI:** 10.3389/fphar.2023.1212634

**Published:** 2023-08-11

**Authors:** Giuseppe Corona, Emanuela Di Gregorio, Angela Buonadonna, Davide Lombardi, Simona Scalone, Agostino Steffan, Gianmaria Miolo

**Affiliations:** ^1^ Immunopathology and Cancer Biomarkers Unit, Centro di Riferimento Oncologico di Aviano (CRO), IRCCS, Aviano, Italy; ^2^ Medical Oncology and Cancer Prevention Unit, Centro di Riferimento Oncologico di Aviano (CRO), IRCCS, Aviano, Italy

**Keywords:** pharmacometabolomics, metabolomics, trabectedin, pharmacokinetics, pharmacodynamics, biomarkers, sarcoma

## Abstract

**Objective:** Trabectedin is an anti-cancer drug commonly used for the treatment of patients with metastatic soft tissue sarcoma (mSTS). Despite its recognized efficacy, significant variability in pharmacological response has been observed among mSTS patients. To address this issue, this pharmacometabolomics study aimed to identify pre-dose plasma metabolomics signatures that can explain individual variations in trabectedin pharmacokinetics and overall clinical response to treatment.

**Methods:** In this study, 40 mSTS patients treated with trabectedin administered by 24 h-intravenous infusion at a dose of 1.5 mg/m^2^ were enrolled. The patients' baseline plasma metabolomics profiles, which included derivatives of amino acids and bile acids, were analyzed using multiple reaction monitoring LC-MS/MS together with their pharmacokinetics profile of trabectedin. Multivariate Partial least squares regression and univariate statistical analyses were utilized to identify correlations between baseline metabolite concentrations and trabectedin pharmacokinetics, while Partial Least Squares-Discriminant Analysis was employed to evaluate associations with clinical response.

**Results:** The multiple regression model, derived from the correlation between the AUC of trabectedin and pre-dose metabolomics, exhibited the best performance by incorporating cystathionine, hemoglobin, taurocholic acid, citrulline, and the phenylalanine/tyrosine ratio. This model demonstrated a bias of 4.6% and a precision of 17.4% in predicting drug AUC, effectively accounting for up to 70% of the inter-individual pharmacokinetic variability. Through the use of Partial least squares-Discriminant Analysis, cystathionine and hemoglobin were identified as specific metabolic signatures that effectively distinguish patients with stable disease from those with progressive disease.

**Conclusions:** The findings from this study provide compelling evidence to support the utilization of pre-dose metabolomics in uncovering the underlying causes of pharmacokinetic variability of trabectedin, as well as facilitating the identification of patients who are most likely to benefit from this treatment.

## Graphical Abstract

**Figure:**
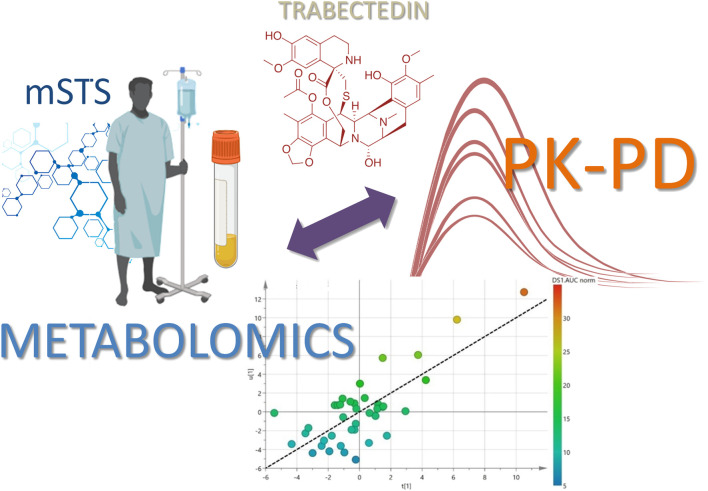

## Introduction

The delivery of the most effective cancer treatment is challenging since it is faced by the management of inter-patient variability due to the complex interplay between drug, host, and tumor. It is well recognized that differences in the pharmacokinetics processes of absorption, distribution, metabolism, and excretion (ADME) as well as environmental and genetic factors significantly contribute to drug response variability. The identification of predictors for drug pharmacokinetics and response is remarkably important for the achieving of personalized treatments especially for cancer therapies where the variability in the exposure to drugs with narrow therapeutic index can lead to over-dosing and side effects or conversely to under-dosing and therapeutic failure.

Numerous studies suggest that pharmacometabolomics may represent a powerful tool to foresee individual drug exposure associated with toxicity development and overall response (Kaddurah-Daouk et al., 2015; Everett, 2016). At the base of pharmacometabolomics is the study of the correlation between metabolome, which represents the complete set of metabolites at a determinate time in a biological system, and the pharmacological effect of drugs (Wishart, 2016; Wishart, 2019). Metabolites, being the downstream products of almost all cellular regulatory processes, incorporate the complex interactions among gene transcription, protein expression, and environmental factors such as age, gender, lifestyle, diet, and physio-pathological conditions. For these characteristics, the metabolome can provide a reliable snapshot of the current individual phenotype, which may embed information about the individual differences potentially responsible for determining the individual drug pharmacokinetics and pharmacodynamics. This concept found wide consensus in different studies where the pharmacometabolomics approach was successfully applied for the identification of predictive biomarkers to optimize drug dosing (Phapale et al., 2010; Huang et al., 2015; Zhang et al., 2017; Kaddurah-Daouk et al., 2018), and to monitor the pharmacodynamic response to treatment. However, only a few of these researches have concerned anticancer drugs (Miolo et al., 2016; Corona et al., 2018; Bao et al., 2019; Wu et al., 2019; Miller et al., 2021), leaving mainly unexplored the application of pharmacometabolomics in this specific field. In an attempt to prove the potential of such a new *omics* tool, the present study focused on the antitumor drug trabectedin to search for metabolomics signatures associated with its high pharmacokinetics and pharmacodynamics variability among mSTS patients. Trabectedin is a tetrahydro-isoquinoline first isolated from the marine tunicate Ecteinascidia turbinata and mainly used as second-line treatment in mSTS patients progressing after anthracycline chemotherapy with higher efficacy in leiomyosarcoma and liposarcoma histotype (L-sarcomas), and translocation related STS (Le Cesne et al., 2012; Kawai et al., 2015). Patients treated with trabectedin report a wide variability in pharmacological response (Demetri et al., 2016), which could be partially ascribed to the high inter-individual drug exposure since its clearance can vary up to about 50% among patients (Perez-Ruixo et al., 2007; Ueda et al., 2014). Thus, both the efficacy and toxicity of trabectedin may take advantage of optimizing individual pharmacokinetics profiles. In this pilot study, we attempt for the first time to elucidate the inter-patient pharmacological variability of trabectedin in STSs patients, achieved through the examination of pre-dose individual plasma metabolomics phenotype. The study results indicate that differences in the individual metabolomics profile among mSTS patients can explain approximately 70% of the pharmacokinetics variability of trabectedin. Moreover, specific pre-dose metabolic signatures were found able to distinguish the patients who receive the most clinical benefit from the treatment. Both findings support the use of metabolomics in the optimization and personalization of trabectedin treatment.

## Materials and methods

### Chemicals

Acetonitrile and methanol (LC-MS grade) were purchased from Carlo Erba Reagents (Milan, Italy). Ultrapure water was generated by a Milli-Q Plus system (Millipore, Billerica, MA, United States). Formic acid, ammonium formate, ammonium acetate, and dimethyl sulfoxide (DMSO) were purchased from Sigma-Aldrich (Milan, Italy). Analytical standards of trabectedin and its deuterium-labeled derivative d3-trabectedin were provided by PharmaMar (Colmenar Viejo, Madrid, Spain). Trabectedin-free human plasma used for calibration curves and quality control preparation was obtained from healthy volunteers at Centro di Riferimento Oncologico, Aviano. The AbsoluteIDQ^®^ Bile Acids (BAs) kit, consisting of five calibrators, three levels of quality controls (QCs), and labeled internal standard (IS), was acquired from Biocrates Life Sciences (Innsbruck, Austria). Analytical reference standards and labeled IS for amino acids (AAs) quantification were purchased from Toronto Research Chemicals (North York, ON, Canada) and Cambridge Isotope Laboratories (Tewksbury, MA, United States of America).

### Study design and patients’ population

The present monocentric study enrolled 40 mSTS patients undergoing trabectedin treatment. The study aimed to find a regression model, based on pre-dose plasma targeted metabolomics profile, able to explain the trabectedin pharmacokinetics variability and to find metabolomics signatures associated with the clinical benefit of the treatment. This latter was assessed at the third chemotherapy cycle identifying the proportion of subjects with responsive disease (RD), stable disease (SD), or progressive disease (PD). All the mSTS patients were enrolled at the Centro di Riferimento Oncologico (CRO) National Cancer Institute, Aviano (Italy). Trabectedin was administered by 24 h-intravenous infusion at a dose of 1.5 mg/m^2^ body surface area every 21 days, after premedication with 20 mg dexamethasone. Normal hematological, renal, and liver functions, as well as the PS ≤ 2, were requested to receive trabectedin therapy together with a 3-week free interval from previous chemotherapy. All patients were evaluated for response to the trabectedin treatment according to Response Evaluation Criteria in Solid Tumours (RECIST). Both plasma metabolomics and clinical variables were included in the development of the best correlation models predictive for PK and PD of trabectedin. The investigation was carried out in accordance with the principles of the Declaration of Helsinki and approved by the CRO Institutional Ethical Committee (nos. 2015.004CE, 09/04/2015, NCT04394728). All subjects gave written informed consent.

### Sample collection

Pre-dose fasting whole blood sample was collected for metabolomics analyses while serial blood sampling was performed for trabectedin pharmacokinetics analysis. Blood sample collection was performed in 5 ml tubes containing EDTA as an anticoagulant at the following time points 0 (pre-dose) and at 2, 4, 8, 24, 25, 28, 32, and 48 h from the start of infusion. Plasma was separated from blood cells by centrifugation at 4°C for 10 min at 3,200 rpm by Heraeus Megafuge 16R centrifuge then samples were immediately transferred into polypropylene tubes and stored at −80°C until analysis.

### Pharmacokinetics analysis

The quantification of plasma trabectedin concentration was carried out using a previously described LC-MS/MS validated method (Di Gregorio et al., 2020). Briefly, 50 μL of plasma samples, calibrators, or QCs were mixed with 200 μL of acetonitrile-1% formic acid containing 0.1 ng/mL trabecedin-d3 as IS. After vortexing and centrifugation at 20,800 g for 10 min at 4°C the supernatant was directly transferred into an auto-sampler glass vial and 3 μL were injected into the LC-MS/MS system constituted by an Ultivo triple quadrupole mass spectrometer (Agilent, Santa Clara CA, United States). The multiple reaction monitoring (MRM) transitions used for quantification were 762 → 234 (m/z) for trabectedin and 765 → 234 (m/z) for the IS. Eight point calibration curves from 0.01 ng/mL LLOQ to 2.5 ng/ml and three QCs sample levels (0.04, 0.8, 0.16 ng/ml) were included in each analytical run. Intra and inter-day precision and accuracy were less than 15%.

The pharmacokinetics parameters were obtained from the non-compartmental model, using the trapezoidal method for the AUC up to 48 h (AUC0-48 h) and extrapolating the C_max_ from the concentration-time plot pharmacokinetics profiles using PC-NONLIN program (version 2.0). Both AUC and C_max_ values were normalized by the respective absolute dose in mg of trabectedin administered to each patient.

### Targeted metabolomics profile analysis

Metabolomics profile was carried out in pre-dose plasma of patients and targeted to 48 AAs and 16 BAs derivatives **(** Supplementary Tables S1, S2). The two classes of metabolites were analyzed separately by LC-MS/MS using a HILIC and reverse phase (RP) chromatographic methods, according to the manufacturer’s instruction Jasem AAs (SRA Instruments Analytical Solutions, Italy) and Biocrates BAs (Innsbruck, Austria) LC-MS/MS kits, respectively. Quantitative analysis was performed by Ultivo triple quadrupole mass spectrometer using internal standard calibration and MassHunter software (Agilent, Santa Clara CA, United States). Intra-assay and inter-assay variability for both AAs and BAs metabolites were ≤15%.

### Statistical analyses

Quantitative metabolomics data were pre-processed by log transformation and unit variance scaling before statistical analyses. Correlations between pharmacokinetics parameters and metabolites plasma concentration were assessed by double-stage multivariate Partial least squares (PLS) analysis, selecting in each step the metabolites with Variable Importance in Projection (VIP) ≥ 1. The internal validation of the model was tested by K-fold cross-validation and by permutation test to assess the degree of overfitting. The Q^2^ and R^2^ value were used as indicators of the predictive ability and goodness of fit model, respectively. Multiple regression analysis was applied after double-stage PLS to further refine the model. The backward method was used to reduce the number of metabolites in the pharmacokinetics predictive model. The multicollinearity of highly correlated variables was checked by evaluation of Variance Inflation Factor (VIF) < 2. Multivariate supervised PLS discriminant analysis (PLS-DA) was used to find differences in baseline metabolomics profile between PD and SD patients as well as to disclose association with the toxicity development following trabectedin treatment. VIP and Student’s t-test were used to identify significantly different plasma metabolite concentrations between groups. Differences in categorical variables were evaluated by Fisher’s exact test, while Pearson correlation was used for continuous variables. The *p*-values were adjusted (q-value) by multiple hypothesis testing based on False Discovery Rate (FDR). Statistical significance was accepted for q ≤ 0.05 unless otherwise specified. Receiver operating characteristic (ROC) analyses were applied to evaluate the ability of selected metabolites to discriminate the groups of interest. Statistical analyses were performed using SIMCA Umetrics version 14.0, MedCalc v. 19.2.1, and MetaboAnalyst 4.0 tools.

## Results

### Patients population

The 40 mSTS enrolled patients had a median age of 66 years with a superimposable percentage of males and females (45% females and 55% males) (Table 1). L-sarcomas were the most prevalent histological subtypes accounting for 42.5% of the cases. The largest portion of the mSTS patients presented poorly differentiated G3 grade tumors, with only 25% of patients that showed moderate G2 grade differentiation. 52.5% of patients showed a good general health condition, with an ECOG performance status (PS) of 0, while the remaining 47.5% had a PS between 1 and 2. Patients received trabectedin for a median of three cycles (range: 1–48) mainly as second-third line (60%), preceded by chemotherapeutic treatments based on anthracyclines or gemcitabine. Trabectedin treatment induced modest hematological toxicities (G0-2), such as anemia, leukopenia, and neutropenia in 60% of patients while G3 side effects were observed in 40% of the patients. Total toxicity (hematological and extra-hematological toxicities) with G0-2 and G3 grades occurred in 52.5% and 47.5% of patients, respectively. In 40% of patients who experienced adverse effects during the first cycle, the dose of trabectedin was managed by a 75% dose reduction administered during the following chemotherapy cycles. After three cycles of treatment, the status of the disease was re-evaluated by computed tomography scan: 20 patients reported a PD, while in 16 patients an SD was achieved. Four patients died before the conclusion of the first three chemotherapy cycles and were not evaluable for the pharmacological response. The median overall survival was 13.2 months (range: 0.8–56.9 months).

**TABLE 1:** Demographic and clinical characteristics of patients.

Characteristics	Value
Sex, n (%)	
Female	18 (45.0)
Male	22 (55.0)
Age (years), median, range	66 (37–90)
Age, n (%)	
<65 years	18 (45.0)
≥65 years	22 (55.0)
BMI (kg/m2), median (range)	27.0 (17.5–41.8)
Tumor subtype, n (%)	
L-sarcomas^a^	17 (42.5)
Other sarcomas^b^	23 (57.5)
Tumour grade, n (%)	
G2	10 (25.0)
G3	30 (75.0)
Performance status (ECOG score), n (%)	
0	21 (52.5)
1–2	19 (47.5)
Trabectedin therapy, n (%)	
1st line	16 (40)
2nd line	18 (45)
3rd line	6 (15)
Hematological toxicity grade, n (%)	
G0-2	24 (60.0)
G3	16 (40.0)
Non-Hematological toxicity grade, n (%)	
G0-2	21 (52.5)
G3	19 (47.5)
Disease status 3rd cycle, n (%)	
PD	20 (50)
SD	16 (40)
NE	4 (10)
Overall survival, median in months (range)	13.2 (0.8–56.9)

BMI, body mass index; ECOG, eastern cooperative oncology group; PD, progressive disease; SD, stable disease; NE, not evaluable.

^a^ Leiomyosarcoma (n = 12) and liposarcoma (n = 5).

^b^ Other sarcomas include: malignant peripheral nerve sheath tumour (n = 3), fibrosarcoma (n = 4), undifferentiated pleomorphic sarcoma (n = 4), chondrosarcoma (n = 2), synovial sarcoma (n = 2), not otherwise specified sarcoma (n = 4), endometrial stromal sarcoma (n = 2), desmoplastic small-round-cell tumour (n = 1) and malignant fibrohistiocytoma (n = 1).

### Pharmacokinetics

**FIGURE 1:**
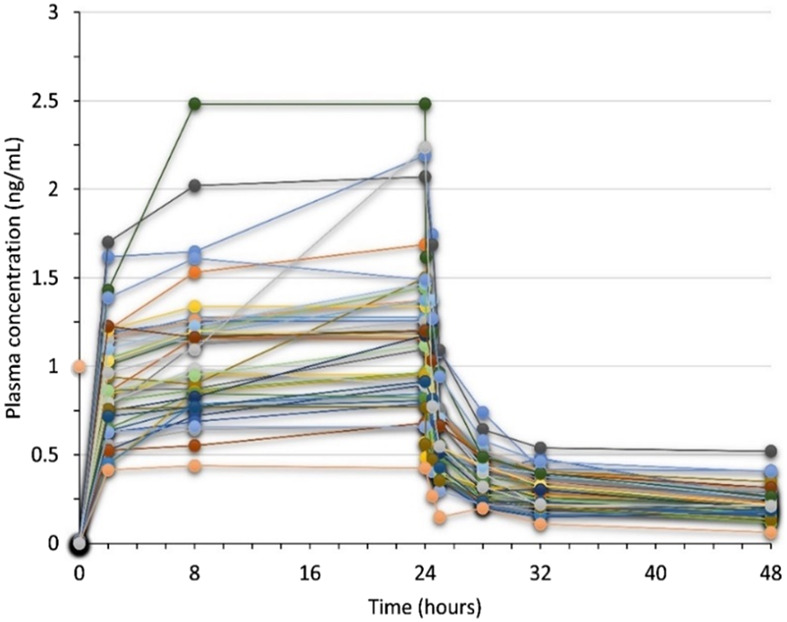
Plasma-concentration time profile of trabectedin in 40 mSTS patients receiving 1.5 mg/m^2^ dose of trabectedin administered as 24 h intravenous infusion.

**FIGURE 2:**
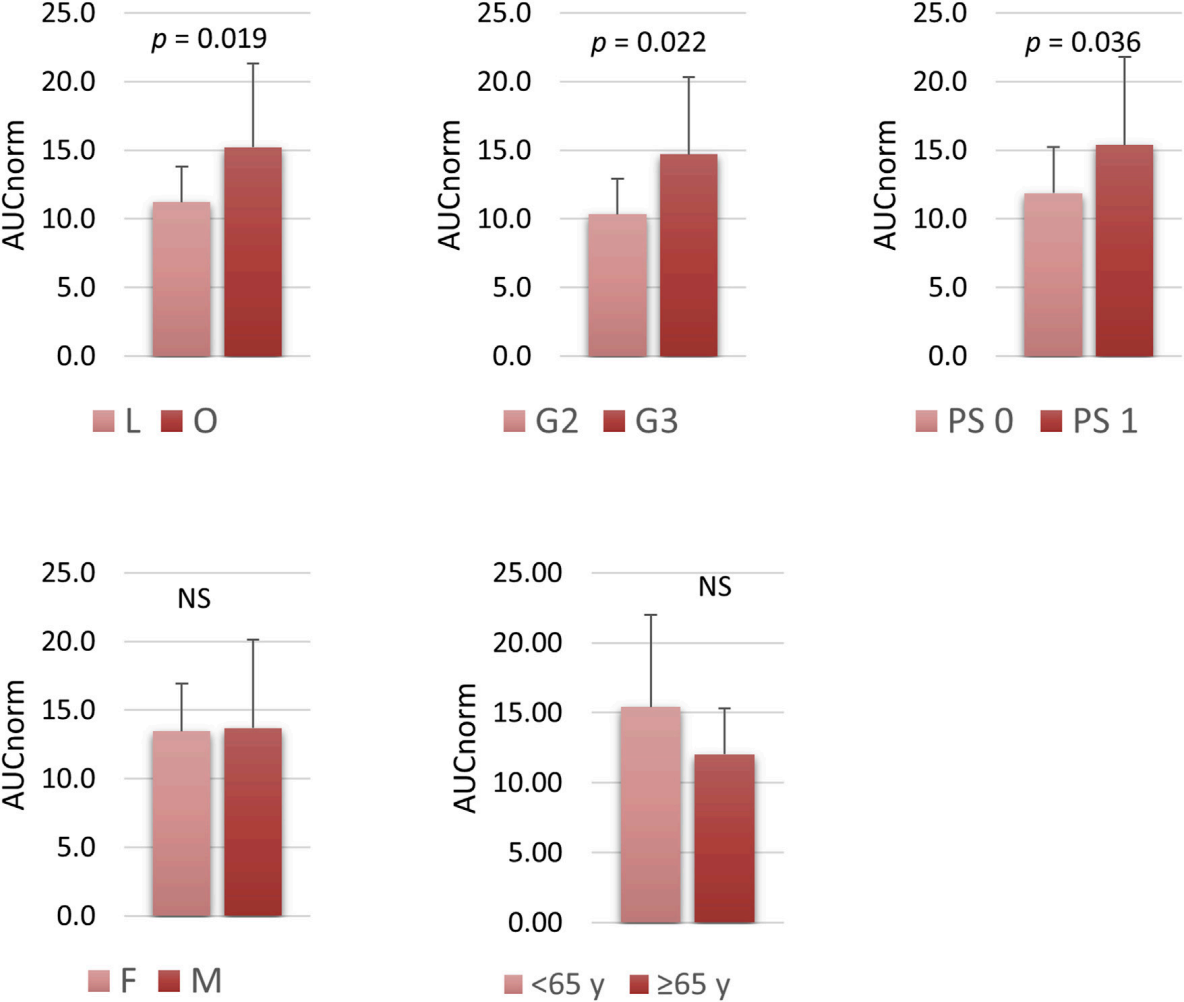
Association between trabectedin AUC and clinical characteristics such as histotypes, tumour grading, PS, sex and age. AUC data are expressed as mean and SD. Age ≥65, PS 0 vs. 1–2. L, leimyosarcoma and lipoisarcoma; O, other sarcomas; PS, performance status; F, female; M, male; G2, G3, tumour grade. AUC normalize by total administered dose.

Trabectedin plasma concentrations were evaluated up to 48 h from the start of administration in all enrolled patients. Throughout the 24-h infusion the trabectedin concentrations increased non-linearly, reaching a steady state at about 8 h from the start of the infusion. The elimination of trabectedin occurred by a bi-exponential kinetics, with a rapid decline immediately after the end of the infusion followed by a slower late elimination phase up to 48 h (Figure 1). The pharmacokinetics parameters of trabectedin are summarized in Table 2. The mean experimental AUC_0-48_ and C_max_ were 33.2 ± 11.2 ngh·mL^-1^ (range: 12.7–63.4) and 1.2 ng mL^-1^ (range: 0.4–2.5) respectively, while AUMC_0-48_ and MRT were 596.8 ± 202.2 ng h^2^ mL^-1^ and 18.0 ± 1.0 h, respectively. The mean AUC normalized by the absolute dose of trabectedin (AUC/Dose) was 13.6 ± 5.31 ng h·mL^-1^mg^-1^ (range 6.33–31.70 ng h·mL^-1^mg^-1^). Differences in the trabectedin AUC/Dose were investigated also as a function of the baseline patients’ clinical and demographic characteristics (Figure 2). Patients with L-sarcomas showed significantly lower AUC/Dose compared with those having other sarcomas (*p* = 0.019). The same trend was observed for the tumor grade: patients carrying grade 3 sarcoma had 1.4-fold higher AUC/Dose compared with those having grade 2 (*p* = 0.022). PS was also found associated with pharmacokinetics since patients with PS = 0 had significantly lower AUC/Dose than those with PS = 1 (*p* = 0.036). No correlations were instead found between pharmacokinetics and gender or age.

**TABLE 2:** Pharmacokinetics parameters of trabectedin.

PK parameters	Mean	SD	Range	95% CI
Cmax (ng·mL^-1^)	1.2	0.5	0.4–2.5	1.1 to 1.4
AUC 0–48 h (ng·h·mL^-1^)	33.2	11.2	12.7–63.4	29.6 to 36.7
AUMC 0–48 h (ng·h^2^·mL^-1^)	596.8	202.2	214.2–1105.1	53 to 660
MRT (h)	18.0	1.0	13.9–20.4	17.7 to 18.3
AUC/Dose (ng·h·mL^-1^·mg^-1^)	13.6	5.31	6.33–31.70	11.9 to 15.3
Cmax/Dose (ng·mL^-1^·mg^-1^)	0.50	0.21	0.22–1.24	0.43 to 0.57

SD, standard deviation; CI, confidence interval; AUC, area under the curve; AUMC, area under the first moment curve; MRT, mean residence time; Cmax; maximum concentration, Dose; absolute dose of trabectedin administered expressed in mg.

### Metabolomics and pharmacokinetics correlations

**FIGURE 3:**
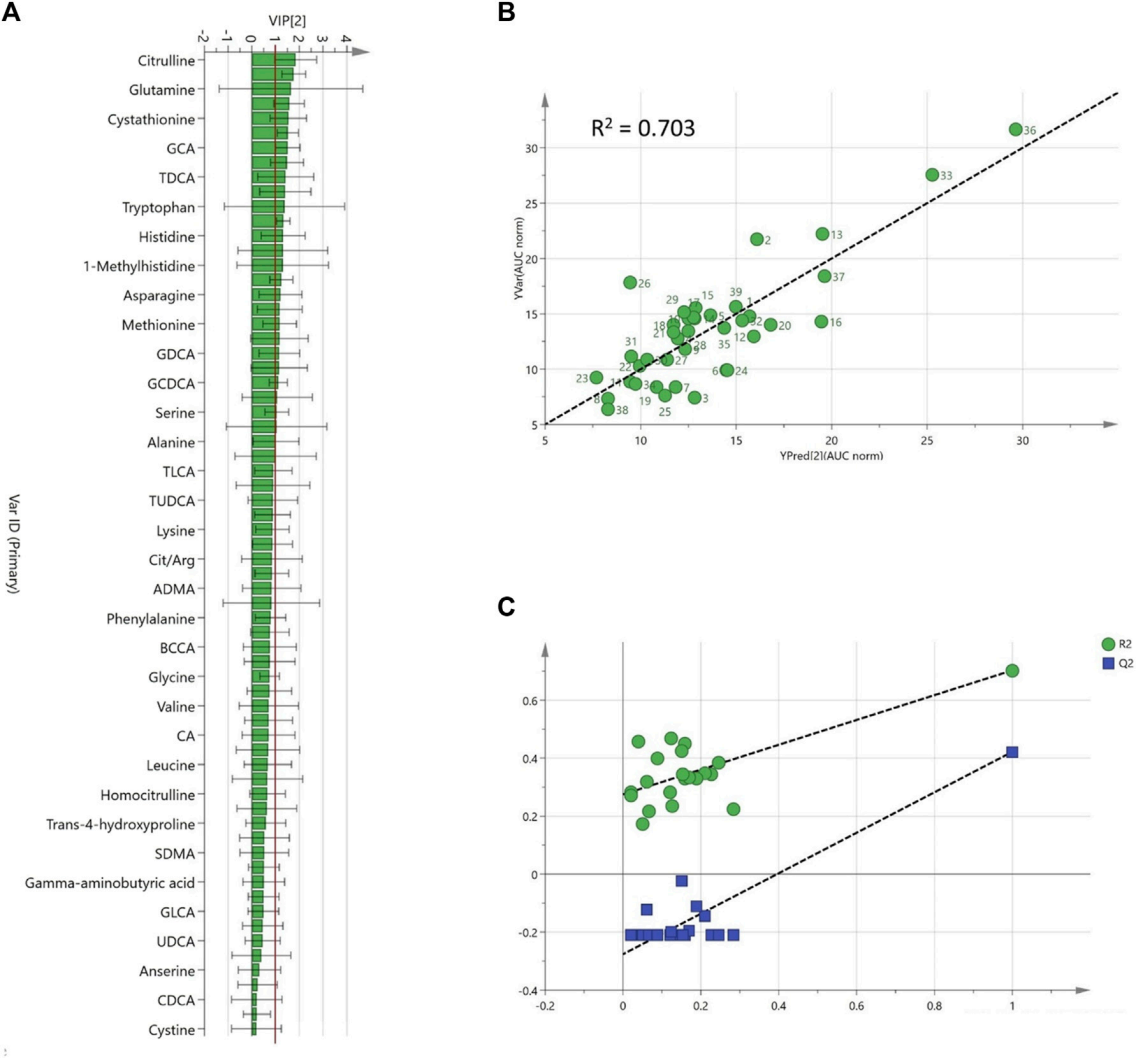
Refined PLS model based on pre-dose plasma metabolites **(A)**; Variable importance projection (VIP) scores ranked, **(B)**; Predicted vs. observed AUC/Dose plot, **(C)**; internal validation goodness of fit (R^2^, green) and predictability parameters (Q^2^, blue) from the permutation analysis.

A predictive model for AUC/Dose trabectedin was developed from the baseline metabolomics profile data by double-stage PLS regression analysis. Before analysis, principal component analysis (PCA) was performed to detect any potential outliers. One patient with an abnormal metabolomics profile was excluded from further analyses (Supplementary Figure S1). An explorative PLS plot (n = 39) of the individual targeted metabolomics profiles showed a good linear relationship with trabectedin AUC/Dose with an R^2^ = 0.59 (Supplementary Figures S2A–C). The refined PLS model, obtained by reducing the noise variables based on only twenty-four metabolites with VIP ≥1, improved fit performance (R^2^ = 0.70) and predictability (Q^2^ = 0.42) (Figures 3A,B). Random permutation test revealed no data overfitting with all R^2^ and Q^2^ values for each permutation lower than the original values (intercepts: R^2^ = 0.275, Q^2^ = −0.276) (Figure 3C). To improve feasibility the predictive model was simplified by backward multiple regression analysis of the metabolomics hits selecting five variables: cystathionine, hemoglobin (Hb), taurocholic acid (TCA), citrulline, phenylalanine-tyrosine (Phe/Tyr) ratio, and that have an independent impact on AUC/Dose prediction without risk of multicollinearity (VIF <2) (Supplementary Table S3). The final multiple regression model: 

AUC/Dose = 20.07–0.15 [Citrulline]-0.82 [Hb]+15.70 [TCA]+2.69 [Cystathionine]+5.16 [Phe/Tyr], still showed a good prediction ability (R^2^ = 0.69) with a bias and precision of 4.6% and 17.4%, respectively (Supplementary Figure S3).

### Metabolomics biomarkers of trabectedin response

**FIGURE 4:**
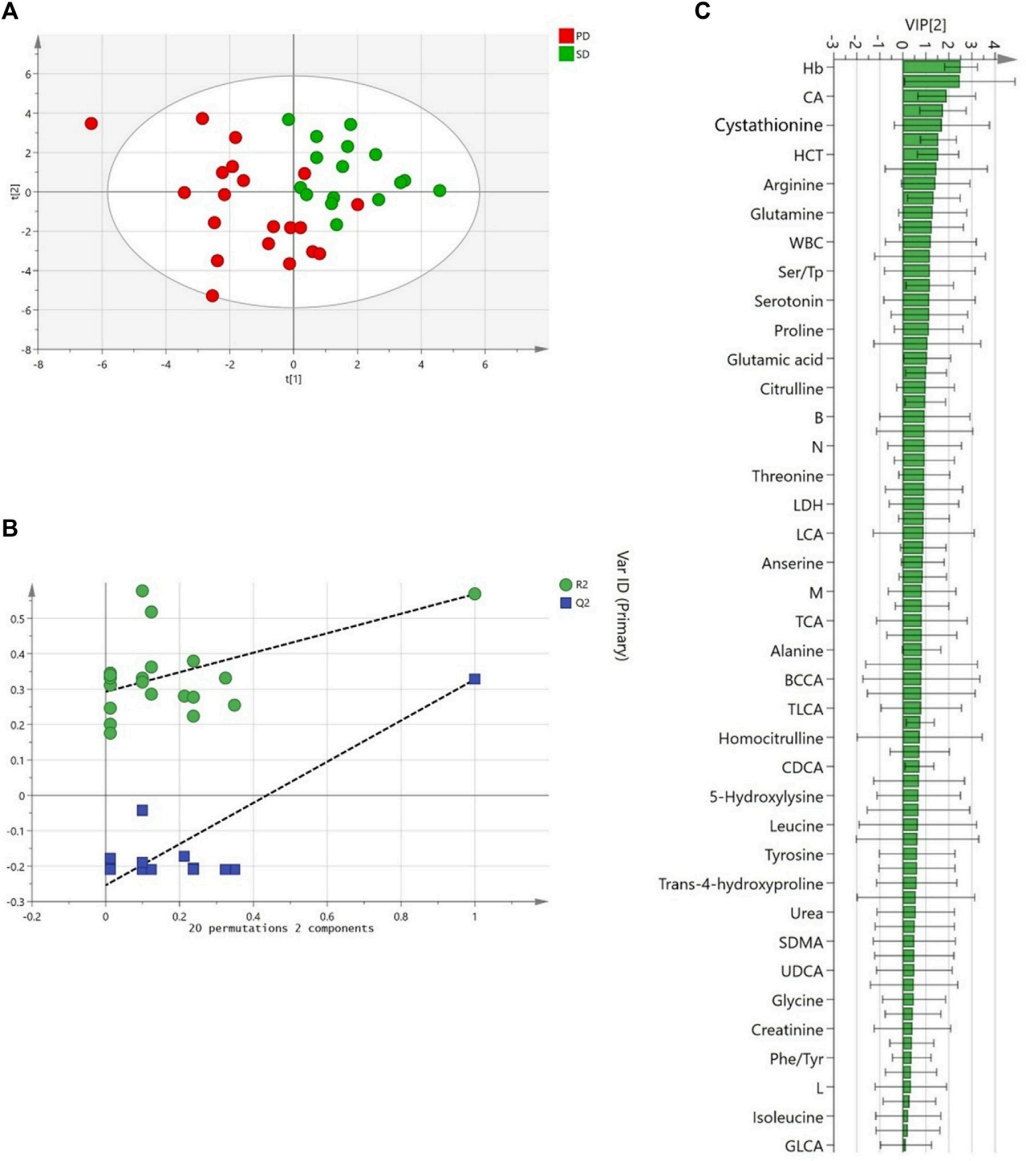
Partial least squares discriminant analysis (PLS-DA) score plot discriminated serum clinical-metabolomics profiles of PD (n = 20, red) and SD patients (n = 16, green) **(A)**. Permutation test showed R2 (green) and Q2 (blue) validation parameters significantly different between permuted and original models **(B)**. Variable importance in projection (VIP) of PLS-DA model ranked by increasing values **(C)**.

**FIGURE 5:**
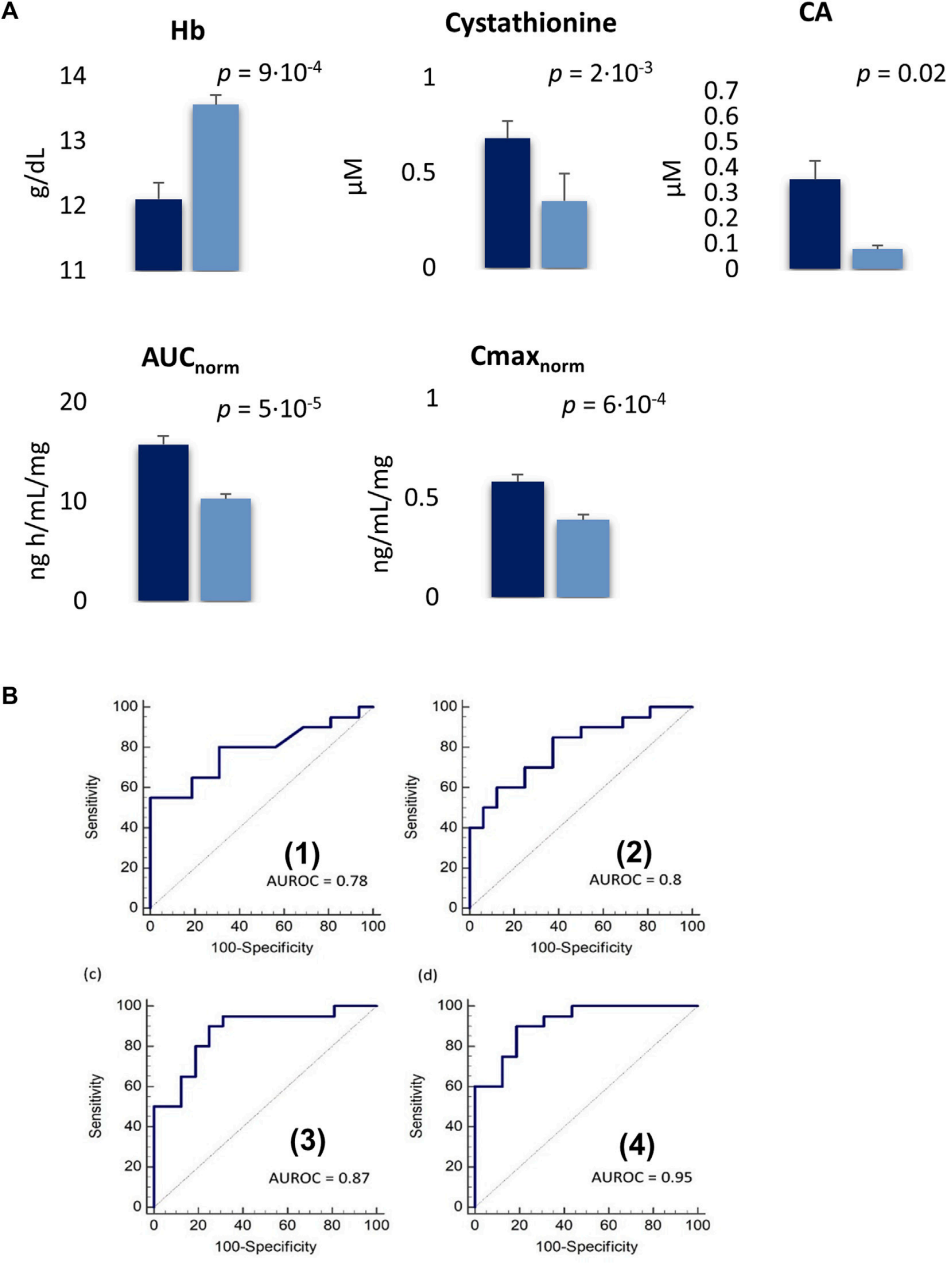
**(A)**; Plasma concentrations of variables significantly different between PD (blue) SD patients (dark blue) expressed as mean ± SE. SE: standard error. **(B)**; Receiver operating characteristic (ROC) curve for single Hb **(1)** and cystahionine **(2)** with an AUROC of 0.78 and 0.8, respectively. The combination of Hb and cystahionone **(3)** and the integration of this latter model with C_max/Dose_ **(4)** achieved an AUROC of 0.87 (90% sensitivity, 75% specificity) and 0.95 (90% sensitivity, 81.3% specificity), respectively.

After three cycles of treatment, 16 patients (40%) achieved an SD, while in the remaining 20 patients (50%) a PD was reported. Four patients (10%) including the metabolomics outlier died before the third cycle and were not included in the analysis. The PLS-DA, incorporating the clinical data, clearly distinguished the baseline plasma metabolomics profile of the SD and PD groups. The model showed an acceptable classification performance (R^2^ = 0.57, Q^2^ = 0.33) without the risk of overfitting. The plasma metabolites variables that mainly contributed to such group classification with a VIP≥1 included 15 AAs derivatives, 3 BAs, and 5 clinical parameters such as Hb; red blood cells (RBC); hematocrit (HCT); white blood cells (WBC); mean cell volume (MCV) (Figure 4A–C). Five variables resulted to be statistically different among SD and PD groups by T-test. In particular, Hb was significantly lower in PD patients (*p* = 9·10^−4^), while plasma cystathionine (*p* = 2·10^−3^) and cholic acid (CA) (*p* = 0.02) were higher in PD patients (Figure 5A). Interestingly, the PD group showed an AUC/Dose of trabectedin 1.5-fold higher than the SD group (*p* = 5·10^−5^). Moreover, high tumor grade and histotypes different from L-sarcomas were found also associated with a poor response (*p* = 0.0017 and *p* = 0.041, respectively) while no significant differences can be referred to PS, age, or gender. Despite the increased AUC/Dose, the toxicity rate did not result statistically different between the SD and PD groups (*p* = 0.503).

The ROC analysis showed Hb and cystathionine the highest diagnostic capability in the classification of SD and PD patients with an AUROC of 0.78 (55% sensitivity, 100% specificity) for Hb and 0.80 (85% sensitivity, 62.5% specificity) for cystathionine, respectively while their combination improves the discriminatory power with an AUROC = 0.87 (sensitivity 90%, specificity 75%) (Figure 5B). Moreover, the integration of the model with the pharmacokinetics parameter C_max_/Dose further enhanced its ability in distinguishing SD *versus* PD patients (AUROC = 0.95, 90% sensitivity, 81.3% specificity) (Figure 5B).

Baseline metabolomics profiles investigated in the context of the hematological and non-hematological toxicity development by PLS-DA analysis showed only a modest separation between the toxicity groups G0-2 (n = 21) or G3 (n = 19) (Supplementary Figure S5). However, the model did not pass the internal cross-validation (R^2^ = 0.47, Q^2^ = −0.21), not allowing the identification of specific metabolomics signatures responsible for trabectedin side effects occurrence. Moreover, the extent of both hematological and non-hematological toxicity observed among patients was not found significantly associated with the pharmacokinetics AUC/Dose of trabectedin (*p* = 0.550).

## Discussion

Trabectedin is effective in the treatment of locally advanced or mSTS patients, although a broad variability in the clinical outcome has been observed (Le Cesne et al., 2015; Demetri et al., 2016). The reasons behind such pharmacological variability are largely unknown, posing a challenge in predicting the clinical benefit of the treatment and emphasizing the need for predictive markers of trabectedin efficacy.

The pharmacokinetics profiles of trabectedin observed in this investigation were found superimposable with those reported in other pharmacokinetics investigations (Perez-Ruixo et al., 2007; Grosso et al., 2020). The drug exposure, expressed by AUC, showed a relatively wide variability of about 34%, in agreement with previous studies, not related to age and gender (Mehren et al., 2008; Grosso et al., 2020). However, other important factors such as individual genetics, as well as specific physio-pathological conditions combined with environmental factors, can contribute to influencing the trabectedin pharmacokinetics profile. All this individual information is not easy to determine but can be captured and integrated into circulating metabolomics profile making it an extraordinary tool to investigate and explain the trabectedin pharmacokinetics differences among patients. This new concept was supported by the results of this investigation since the baseline metabolomics profile was found strongly correlated with the AUC of trabectedin demonstrating that individual metabolic features may include information associated with drug clearance. The optimized PLS regression model, based on citrulline, Hb, cystathionine, TCA and Phe/Tyr ratio targeted metabolomics, allowed to predict the individual pharmacokinetics of trabectedin explaining almost 70% of inter-individual variability. These variables do not guarantee specificity to the disease alone, but they can contribute to the overall understanding of a specific pharmacological profile. Although these emerged metabolites are not directly involved in the ADME process, it is well-known that both endogenous and exogenous metabolites can indirectly induce alterations in drug pharmacokinetics by affecting the expression and activity of cytochrome 3A4 (CYP3A4) which is the main enzyme responsible for the hepatic metabolic clearance of many drugs including trabectedin (Vermeir et al., 2009; Machiels et al., 2014). Within this framework, the TCA derivative, which is a primary BA synthesized from cholesterol and conjugated with taurine in the liver, can represent one of such endogenous metabolites. Indeed, BAs, beyond their major activity in dietary lipids absorption, can alter the CYP expression through direct interaction with the nuclear farnesoid X receptor (FXR) (Gnerre et al., 2004). In particular, high levels of primary free and conjugated BAs have been found to enhance CYP3A4 expression to defense cells from their toxic effect (Chen et al., 2014). Conversely to this mechanism, we found a direct positive correlation between TCA levels and AUC. This suggests that TCA may interact through an alternative mechanism that reduces trabectedin clearance. Since the regulation and detoxification of BAs processes involve CYP3A4 (Stedman et al., 2004), high concentrations of TCA may compete for the catalytic site of this specific cytochrome contributing to reducing the trabectedin clearance. This hypothesis appears supported by other studies which report a positive correlation between serum BAs and the AUC of drug metabolized by CYP3A4 (Taegtmeyer et al., 2014; McCune et al., 2023). In addition to metabolic clearance, elevated TCA levels can impact the AUC of trabectedin by modifying its cellular transport and elimination. This is because both trabectedin and BAs are substrates of ATP Binding Cassette 1–2 (ABCC1-2), also referred to as multidrug resistance-associated protein 1–2 (MRP1-2) (Beumer et al., 2010). These proteins, mainly localized in the membrane of hepatocytes, kidney, and intestine cells, regulate the excretion of organic anions like bilirubin, BAs, and xenobiotics protecting cells from their toxic accumulation. Taking into account this transport mechanism, increased TCA levels could function as a competitive antagonist for the ABCC binding of trabectedin. Consequently, this could lead to higher plasma concentrations of trabectedin. Moreover, beyond TCA, also other BAs, such as glycocholic acid, taurodeoxycholic acid, and taurochenodeoxycholic acid, showed a positive correlation with AUC, likely contributing to hamper trabectedin elimination mediated by ABCC. In patients and animals with dysfunctional ABCC transporters the increase in systemic trabectedin exposure was found associated with acute severe hepatotoxicity (Lee et al., 2008; van Waterschoot et al., 2009). Nevertheless, in our patient cohort, we did not identify any noteworthy connections between trabectedin AUC and liver toxicity, likely because all patients received pre-treatment with dexamethasone (Martin et al., 2008; Micuda et al., 2008), a potent ABCC inducer, which effectively can mitigate the risk of severe hepatic injury. Unlike TCA, the other AUC predictors such as cystathionine, citrulline, Phe/Tyr ratio, and Hb, did not show any potential link with the ADME processes. Serum accumulation of cystathionine was observed in patients with aggressive tumors while low serum levels of citrulline were frequently associated with poor outcomes indicating the potential negative prognostic role of these two metabolites (Ouaknine Krief et al., 2019; Miolo et al., 2020). Moreover, the Phe/Tyr ratio, which was proposed as a surrogate metabolic marker of immune activation associated with inflammation, was found elevated in advanced cancer patients that commonly present such immune dysregulation (Neurauter et al., 2008; Ploder et al., 2008). Thus, these metabolites more likely seem to outline specific pathological conditions characteristic of highly aggressive cancer that indirectly predispose to an increased trabectedin exposure. The evidence that individuals with high AUC display a poor performance status, a higher tumor grade, or a more aggressive non-L-sarcoma histotype may further bolster the aforementioned metabolic-pharmacological hypothesis. Beyond pharmacokinetics, the information embedded in the pre-dose metabolomics profiling could also infer trabectedin pharmacodynamics. Indeed, PD patients can be clearly distinguished from those with SD through PLS-DA analysis based on both clinical and metabolomics data. The metabolites that more contributed to the phenotype differentiation were: low Hb and high cystathionine levels all identified as negative prognostic factors. Cystathionine and Hb biomarkers resulted as the best classifiers by ROC analysis, especially when used in combination. The tumor metabolic reprogramming can induce modifications not only limited to the cellular level but also involving the host systemic metabolism. Thus, certain concentrations of circulating metabolites could be depleted to meet cancer’s demands, while others could be synthesized to sustain cancer proliferation and metastasis (Vander Heiden and DeBerardinis, 2017). In this context, cystathionine and Hb may represent a specific metabolomic signature arising from the complex host-tumor metabolic interplay. Cystathionine is an important intermediate of the trans sulphuration pathway which initiates with the homocysteine and serine condensation by the cystathionine β-synthase to produce cystathionine, followed by its hydrolysis into cysteine (Sbodio et al., 2019). The latter is essential for the synthesis of glutathione (GSH), which protects cells from reactive oxygen species (ROS) damage (Belalcázar et al., 2013). The high cystathionine concentrations observed in the PD group might reflect the intensification of the trans-sulfur pathway to sustain the pool of cysteine and GSH, which makes cancer cells able to survive even in highly oxidant environments as those induced by chemotherapeutics (Kennedy et al., 2020). This hypothesis is corroborated by the observation of a high cystathionine β-synthase expression in drug-resistant tumor phenotype (Bhattacharyya et al., 2013; Wang et al., 2018).

The low level of Hb, in general, is indicative of a subclinical anemia status that has been frequently reported as a poor prognosis biomarker for mSTS as well as in other tumor types (Szkandera et al., 2014; Shi et al., 2021). It has been suggested that an inflammatory process, likely induced by tumor itself, can lead to the development of a chronic anemic status (Natalucci et al., 2021). In this scenario, interleukin-6 (IL-6), a pro-inflammatory cytokine, that was often found elevated in advanced cancers including STS (Hagi et al., 2017; Nakamura et al., 2020), can reduce gut iron absorption resulting in blood Hb drop (Nemeth et al., 2004). Moreover, low Hb levels induce tumor hypoxia that, in turn, can trigger the activation of several molecular pathways promoting angiogenesis, anaerobic metabolism, and the transcription of target genes that enhance cell proliferation and tumor metastasis (Lv et al., 2017). On this basis, the low Hb levels that characterize PD patients, as observed in the present study, may indicate a more aggressive tumor behavior that is associated with a poor prognosis. The predictive power for the clinical benefits of trabectedin treatment, as well as its specificity, can be enhanced when information from Hb and cystathionine were integrated with pharmacokinetic parameters such as Cmax which is highly correlated with AUC, providing surrogate information on the overall drug exposure. Its determination requires only a simple blood sample, which makes it useful for enhancing the prognostic clinical model in the clinical setting. However, further investigations are necessary to validate these metabolomics predictive biomarkers, given the small patient cohort and the absence of an external independent group, which limits its full validation.

## Conclusion

The findings of this investigation confirm the potential of metabolomics as a valuable tool for predicting the pharmacokinetics and pharmacodynamics of trabectedin in mSTS patients. This exploratory pharmacometabolomics study represents a significant improvement, into elucidate the inter-patient pharmacokinetic variability of trabectedin by leveraging individual metabolomic characteristics. The study results demonstrate that the baseline plasma metabolomic profile holds significant advantages in revealing differences in individual pharmacological phenotypes which may be associated with both trabectedin exposure and the clinical benefits. These findings highlight the significance of integrating metabolomics into clinical practice, as it can potentially enhance the optimization of treatment in terms of efficacy and safety.

## Data Availability

The raw data supporting the conclusion of this article will be made available by the authors, without undue reservation.
