# Supplementary material for: Pharmacometabolomics of trabectedin in metastatic soft tissue sarcoma patients
Source: Front Pharmacol. 2023 Aug 11;14:1212634. doi: 10.3389/fphar.2023.1212634 (PMC10450632; doi:10.3389/fphar.2023.1212634)
Supplement: Supplementary file 1 [file DataSheet2.PDF]

**Supplemental information**

**Table S1 and S2:** List of targeted amino acid and bile acid derivatives respectively.

**TableS3:** Data of multiple regression analysis for the significant predictive variables.

**Figure S1**: Principal component analysis (PCA) of the pre-dose serum metabolomics profiles of STSs patients.

**Figure S2:** Preliminary PLS regression analysis for $AUC_{/Dose}$ prediction by baseline serum metabolomics profiles. Each dot represents a patient plotted as latent X-score variable, t[1], indicating the individual baseline serum metabolomics data, *vs.* the latent Y-score variable, u[1], representing the AUC/Dose. The colour scale from blue to red indicates increasing values of AUC (a). In the PLS loading plot each point is a metabolite plotted as loading coefficient from PLS LV1 (w*c1) *vs*. the coefficient from LV2 (w*c2) where the metabolites having a positive relationship with the $AUC_{/Dose}$ were those with high positive coefficients placed in the top right quadrant, conversely, those inversely correlated with the $AUC_{/dose}$ were depicted in the bottom left with negative coefficients (b). List of the serum metabolites mostly correlated with AUC sorted by decreasing loading coefficients (c).

**Figure S3:** Predicted vs. observed AUC/Dose plot from model derived by multiple regression analysis.

**Figure S4:** PLS-DA score plot based on serum clinical-metabolomics profiles of patients separated for G0-2 (*n* = 21, green) and G3 toxicity (*n* = 19, blue).
