# Supplementary material for: Pharmacometabolomics of trabectedin in metastatic soft tissue sarcoma patients
Source: Front Pharmacol. 2023 Aug 11;14:1212634. doi: 10.3389/fphar.2023.1212634 (PMC10450632; doi:10.3389/fphar.2023.1212634)
Supplement: Supplementary file 2 [file DataSheet4.PDF]

Table S2. List of targeted bile acids derivatives.

n	Name
1	Cholic acid (CA)
2	Chenodeoxycholic acid (CDCA)
3	Deoxycholic acid (DCA)
4	Glycocholic acid (GCA)
5	Glycochenodeoxycholic acid (GCDCA)
6	Glycodeoxycholic acid (GDCA)
7	Glycolithocholic acid (GLCA)
8	Glycoursodeoxycholic acid (GUDCA)
9	Hyodeoxycholic acid (HDCA)
10	Taurocholic acid (TCA)
11	Taurochenodeoxycholic acid (TCDCA)
12	Taurodeoxycholic acid (TDCA)
13	Taurolithocholic acid (TLCA)
14	Tauroursodeoxycholic acid (TUDCA)
15	Ursodeoxycholic acid (UDCA)
16	Chenodeoxycholic acid (CDCA)
