# Supplementary material for: Pharmacometabolomics of trabectedin in metastatic soft tissue sarcoma patients
Source: Front Pharmacol. 2023 Aug 11;14:1212634. doi: 10.3389/fphar.2023.1212634 (PMC10450632; doi:10.3389/fphar.2023.1212634)
Supplement: Supplementary file 7 [file DataSheet3.PDF]

Table S1. List of targeted aminoacid derivatives.

n	Name	n	Name
1	1-Methyl-L-Histidine	27	Isoleucine
2	2-Aminoadipic acid	28	Anserine
3	2-Aminobutyric acid	29	Leucine
4	3-Aminoisobutyric acid	30	Lysine
5	3-Methyl-L-Histidine	31	Methionine
6	5-Hydroxylysine	32	Ornithine
7	ADMA	33	ortho-Phosphoserine
8	Alanine	34	Phosphorylethanolamine
9	Arginine	35	Phenylalanine
10	Argininosuccinic acid	36	Proline
11	Asparagine	37	Sarcosine
12	Aspartic acid	38	SDMA
13	β-Alanine	39	Serine
14	Carnosine	40	Serotonin
15	Citrulline	41	Taurine
16	Creatinine	42	Threonine
17	Cystathionine	43	trans-4-Hydroxyproline
18	Cystine	44	Tryptophan
19	Ethanolamine	45	Tyrosine
20	γ-Aminobutyric acid	46	Urea
21	Glutamic acid	47	Valine
22	Glutamine	48	Kynurenine
23	Glycine		
24	Histidine		
25	Homocitrulline		
26	Homocystine		
