# Supplementary material for: Pharmacometabolomics of trabectedin in metastatic soft tissue sarcoma patients
Source: Front Pharmacol. 2023 Aug 11;14:1212634. doi: 10.3389/fphar.2023.1212634 (PMC10450632; doi:10.3389/fphar.2023.1212634)
Supplement: Supplementary file 9 [file DataSheet5.PDF]

Table S3. Multiple regression analysis of significant predictive variables.

Independent variables	Coefficient	p-value	$r_{partial}$	VIF
Cystathionine	2.69	0.036	0.35	1.44
Hb	-0.82	0.037	-0.35	1.56
TCA	15.70	0.001	0.53	1.62
Citrulline	-0.15	0.011	-0.42	1.59
Phe/Tyr	5.16	0.013	0.42	1.07
(Constant)	20.07			

VIF, Variance Inflation Factor; $r_{partial}$, partial correlation coefficient.
