# Supplementary material for: Pharmacometabolomics of trabectedin in metastatic soft tissue sarcoma patients
Source: Front Pharmacol. 2023 Aug 11;14:1212634. doi: 10.3389/fphar.2023.1212634 (PMC10450632; doi:10.3389/fphar.2023.1212634)
Supplement: Supplementary file 8 [file DataSheet1.PDF]

CRO
AVIANO

# CENTRO DI RIFERIMENTO ONCOLOGICO

## ISTITUTO NAZIONALE TUMORI - AVIANO

ISTITUTO DI RICOVERO E CURA A CARATTERE SCIENTIFICO DI DIRITTO PUBBLICO (D.I. 31/07/1990)

Via Franco gallini, 2 - 33081 Aviano (PN) Italy - C.F.-P.I. 00623340932 - Tel. 0039-0434-659111 - Fax: 0039 0434 652182

Immunopathology and Cancer Biomarkers Unit – Department of Translational Research

April 26th, 2023

To Prof. Heike Wulff PhD
Editor-in-Chief of Frontiers in Pharmacology

Dear Editor,

I would like to submit, on behalf of all authors, the manuscript entitled "*Pharmacometabolomics of Trabectedin In Metastatic Soft Tissue Sarcoma Patients* " to be considered for publication in the special issue " Pharmacometabolomics: An Omics Approach Towards the Identification of Novel Therapeutic Targets for Personalized Medicineas research article in Clinical Pharmacology & Therapeutics".

In this study, we aimed to investigate whether differences in pre-dose metabolomics profile among metastatic soft tissue sarcoma (mSTS) patients can be associated with the pharmacokinetics and pharmacodynamic variability of trabectedin in mSTS treatment. Our results indicate that pre-dose metabolomics profile, which integrates specific individual physio-pathological conditions and environmental factors, may effectively contain metabolic signatures that can predict both exposure and response to trabectedin.

We believe that the use of pre-dose metabolomics profile analysis in clinical settings may represent a valuable and complementary tool in improving the selection of mSTS patients who receive the best clinical benefit from trabectedin treatment.

We hope that our study will contribute to the development of personalized medicine strategies for mSTS patients and advance the field of pharmacometabolomics.

Each of the authors confirms that this manuscript is original, it is not been previously published and it is not currently under consideration by any other journal. Additionally, all of the authors have approved the contents of the manuscript and they have agreed to the submission.

We hope that the contents and form of the paper will be suitable for publication in Clinical Pharmacology & Therapeutics journal

Thank you for your consideration of this manuscript.

Sincerely,

Giuseppe Corona PhD
Immunophatology and Cancer Biomarkers Unit
Department of Translational Research IRCCS-National Cancer Institute
Via Franco Gallini, 2
33081 Aviano (PN), Italy.
Tel. +39-0434 659666
Fax +39-0434-659659
Email:giuseppe.corona@cro.it
